# Protective Efficacy of Different Live Attenuated Infectious Bronchitis Virus Vaccination Regimes Against Challenge With IBV Variant-2 Circulating in the Middle East

**DOI:** 10.3389/fvets.2019.00341

**Published:** 2019-10-09

**Authors:** Hesham A. Sultan, Ahmed Ali, Wael K. El Feil, Abdel Hamid I. Bazid, Mohamed A. Zain El-Abideen, Walid H. Kilany

**Affiliations:** ^1^Birds and Rabbit Diseases Department, Faculty of Veterinary Medicine, Sadat City University, Sadat City, Egypt; ^2^Poultry Diseases Department, Faculty of Veterinary Medicine, Beni-Suef University, Beni-Suef, Egypt; ^3^Poultry Diseases Department, Faculty of Veterinary Medicine, Suez Canal University, Ismailia, Egypt; ^4^Virology Department, Faculty of Veterinary Medicine, Sadat City University, Sadat City, Egypt; ^5^Reference Laboratory for Veterinary Quality Control on Poultry Production, Animal Health Research Institute, Giza, Egypt

**Keywords:** IBV, live attenuated vaccine, variant-2, vaccination regimes, Middle East

## Abstract

Six vaccination regimes using classical (Mass-type) and variant (IB-VAR2 and IB-793B) live vaccines were evaluated against Middle Eastern variant-2 infectious bronchitis virus challenge. Six groups of SPF chicks (30 birds/group) were vaccinated using prime-boost regimes at day-1 and day-14 using; IB-M41:IB-VAR2, IB-VAR2:IB-VAR2, IB-VAR2:IB-M41, IB-Ma5:IB-793B, IB-793B:IB-793B, and IB-793B:IB-Ma5, respectively. Ciliostasis and lesion scores were evaluated at day-5 after each vaccination. Birds were challenged intranasally at 14-day post 2nd vaccination using 10^5^EID_50_/0.1 ml/bird of wild-type IBV (Eg/1212B/2012). At 3, 5, and 7-day post challenge (DPC) virus shedding was monitored by real-time RT-PCR. Five chicks/group were euthanized at 7DPC for ciliostasis and lesion scoring and histopathology was conducted on 3 chicks/group. Seroconversion was evaluated at 14 DPC. All groups primed with the 793B vaccine showed relatively higher ciliostasis scores compared to other groups. The IB-VAR2 vaccinated groups showed the highest protection rates (80–100%) and high protection score (67.6–73.2%) compared to the 793B vaccine groups (50–60%). The virus shedding was significantly reduced at 3 and 5DPC in groups received the IBV-VAR2 (prime or booster) compared to those received the 793B vaccine. In conclusion, the homologous IBV-VAR2 vaccine showed superior results compared to 793B or Mass-type vaccines confirming the importance of IBV vaccine seed homology to the circulating IBV strains.

## Introduction

Avian Infectious bronchitis (IB) is a highly contagious and economically important worldwide viral disease of chickens. It affects chickens of all ages with severe signs in younger birds and high mortality rates especially when a co-infection with a secondary bacterial or viral pathogen(s) (1–4). Infectious bronchitis virus (IBV) mainly causes respiratory disease and nephritis in chickens but can also result in poor weight gain and lost feed efficiency in broilers and reduced numbers and quality of eggs in layers (5).

The IBV is a *Gammacoronavirus* belonging to the family *Coronaviridae* (6). IBV is characterized by a high mutation rate resulting in changes in viral genotype, antigenic properties, tissue tropism, pathogenicity and eventually the course of the disease (7). Several IBV serotypes or antigenic variant strains emerged due to changes in the IBV genome through point mutations, deletions, insertions or RNA recombination and these variants are often responsible for IB outbreaks in vaccinated chicken flocks (8–10). Hence, pathogenic variants such as D274 and D1466, 793B, Israel variant 1, and 2 (11, 12) have evolved over the last decades.

Several countries have shown that multiple IBV strains are circulating in their poultry flocks. The IS/885/00 and IS/1494/06 or those with high similarities to these strains of IBVs have been reported throughout the Middle East and North Africa (13), Iraq (14), and Egypt (2, 10). Though new vaccines cannot be developed against every emerging variant. However, new vaccines such as the vaccines based on IBV strain 793B (15), QX-like IB strains (16), or Middle Eastern IB-VAR2 (17) have been developed from these pathogenic strains and showed better protection rates.

Alternatively, the assessment of cross-protection of some vaccine combinations against IBV strains of different serotypes is an alternative approach for IBV control (18–20). Cross-protection between IBV strains can be ranged from very poor to moderate protection according to the results of IBV cross-protection studies (21). Under the field conditions, chickens are exposed to different IBV variant strains at the same time. Therefore, it is important to evaluate different vaccine combination safety and efficacy against the circulating IBV strains (19, 20). A recent study carried out by Terregino et al. (22) where the simultaneous or alternate use of Ma5 and 793B, commonly employed in Europe, induces high levels of protection against heterologous IBV types such as D1466 or QX strains. The broadening of protection was previously attributed to increased cellular and local immune responses at tracheal mucosa after combining different live IBV in vaccination programs (18, 19).

However, protection studies indicated that homologous strain vaccines usually induce better protection against IBV challenge (23–25). The field situation in Egypt indicates that the IBV variant 2 is the most predominant serotype in Egypt (26–29), hence the newly developed vaccines using the variant 2 strain showed better protection againest homologous challenge under both expermintal and field situation (17, 30). The Egyptian variant-2 viruses shows high genetic difference to all IBV imported vaccines with multiple amino acid substitutions at virus neutralization (VN) epitopes (31–33) that may explain the high frequency of IBV outbreaks in vaccinated flocks in Egypt.

This study aimed to evaluate the protective efficacy of 3 different vaccination regimes using combinations of an attenuated Egyptian IBV variant-2 vaccine combined with Egyptian Mass type vaccine in comparison to their corresponding variant 793B and Mass-type live attenuated vaccines against the Middle Eastern IBV variant-2 virus.

## Materials and Methods

### Vaccines and Viruses

Two commercially available live attenuated IBV vaccines, ME VAC IB-VAR2® (IB-VAR2) and ME VAC IB-M41® (IB-M41) (ME VAC, Egypt) produced from two IBV strains isolated from Egypt compared to another 2 commercial IBV vaccines, Nobilis® 4/91 (IB-793B) and Nobilis® IB Ma5 (IB-Ma5) (Intervet International B.V., Boxmeer-Holland). All the vaccines were given according to the manufacturer's recommended doses via the intranasal route. The challenge virus was the wild-type Egyptian variant 2 strain Eg/1212B/2012 (GenBank accession no.: JQ839287). For genetic analysis of different vaccines seed strains, a BLAST search was conducted for each sequence (http://www.ncbi.nlm.nih.gov /BLAST). Sequence comparisons and phylogenetic relationships through a bootstrap of 1,000 trials were determined with the MEGA version X program using the Clustal W alignment algorithm (34).

### Experimental Design

All experiments were conducted according to Animal Research Ethics Guidelines at the Faculty of Veterinary Medicine, Beni-Suef University, Egypt.

Two hundred and forty day-old SPF chickens were obtained from Kom-Osheim SPF project, Fayoum, Egypt. Chicks were divided into 8 groups (30 chicks/group) in separate chicken isolators. Birds were provided with feed and water *ad libitum*. The vaccination regimes were following: IB-M41: IB-VAR2, IB-VAR2: IB-VAR2, IB-VAR2: IB-M41, IB-Ma5: IB-793B, IB-793B: IB-793B, and IB-793B: IB-Ma5 on day-1: day-14, respectively. The last two groups were non-vaccinated challenged (G7) and non-challenged control (G8) (Table 1). Ciliary activity was evaluated at 5 days post vaccination (DPV) (both prime and booster vaccination). At 14 days after the prime and booster vaccination, blood samples were collected and the seroconversion against IBV was evaluated. At 28 DPV, birds were challenged with 10^5^EID_50_/0.2 ml/bird with the wild-type Eg/1212B/2012 variant 2 IBV virus via the ocular route (17). Oro-pharyngeal swabs were collected from challenged chicks at 3, 5, and 7 DPC for quantification of virus shedding. the post challenge ciliary activity was evaluated at 7 DPC, where 5 chicks/group were euthanized. Also, the trachea and kidneys were collected from 3 chicks/group for histopathological examination. At the end of the experiment, blood samples were collected from remaining birds for serological evaluation (Table 1).

**Table 1:** Vaccination schedules, ciliostasis scores, protection scores, and protection % of vaccinated chickens after challenge with the wild type IB variant-2 virus.

**Groups (30 bird/group)**	**Vaccination regime**		**Mean ciliostasis score^a^**	**Protection score^b^**	**Protection %^c^**
	**Prime 1st day**	**Booster 14th day**			
1	IB-M41	IB-Var2	11.5	67.6	100
2	IB-Var2	IB-Var2	9.5	73.2	100
3	IB-Var2	IB-M41	15.5	56.3	80
4	IB-Ma5	IB-793B	17.5	50.7	60
5	IB-793B	IB-793B	21.0	40.8	50
6	IB-793B	IB-Ma5	26.5	25.4	50
7	No vaccine challenge		35.5	0.0	0.0
8	No vaccine No challenge		4.0	NA	NA

a *Mean ciliostasis score/bird for the 5 tracheas examined in each group*.

b $protection\;score=\left(1-\frac{mean\;ciliostsis\;score\;for\;vaccinated\;challenged}{mean\;ciliostsis\;score\;for\;challenged\;group}\right)\times \;100$.

c *% of protected chicks: an individual chick was recorded as protected against challenge if the ciliostasis score <20*.

### Tracheal Ciliary Activity Evaluation

The tracheal ciliary activity was assessed as previously described (35). Briefly, three sections of the upper, middle and lower parts of the trachea were analyzed. The rings were placed in a petri dish containing Minimum Essential Medium (MEM) with 10% fetal bovine serum and examined under inverted light microscope for integrity and the ciliary movement of the tracheal epithelial cells. The average ciliostasis score for each group was calculated and the protection score was calculated using the following formula:

(1) $$\begin{array}{l} protection\;score\\ =\left(1-\frac{\text{mean ciliostsis score for vaccinated challenged}}{\text{mean ciliostsis score for challenged group}}\right)\times \;100 \end{array}$$

### Hemagglutination Inhibition Test (HI)

The IB-M41 and IBV-4/91 refrence strains sera and antigens were purchased from Istituto Zooprofilattico Sperimentale delle Venezie (IZSVe, Italy). The IB-VAR2 antiserum and antigen were prepared from the Eg/1212B/2012 IBV challenge strain (GenBank accession no.: JQ839287) at our laboratory as previously described (30). The IBV HI tests were conducted as previously described (36). Breifly, serum samples were kaolin-treated before testing. Serial 2-fold dilutions of treated serum in PBS were done and to each serum dilution, 25 μl of the diluted IBV homologous antigens of each vaccine used (8 HA units/25 μl) was added separately. After challenge, all groups were tested only againest 8 HA units/25 μl of the IB-VAR2 antigen. The plates were incubated at room temperature for 20 min. then 25 μl of 0.5% chicken RBC's were added to each well, and the plates were mixed and incubated for 40 min at room temperature. The HI titer of a sample was calculated as the reciprocal of the last serum dilution with no HA. Negative control serum and antisera against both classical and variant IBV antigen viruses were included in the test.

### Virus Shedding Titers

The viral RNA was extracted by Bioflux® viral RNA Mini Spin column kit (Bioflux, China) in accordance with manufacturer's instructions. Verso 1-Step qRT-PCR Kit (Thermo Scientific, USA) was used for detection and quantification of S1 gene of IBV. The qRT-PCR reaction volume was 25 μl containing 5 μl of extracted RNA, 12.5 μl 2X One-step RT-PCR ready mix, 1.25 μl RT enhancer, 0.25 μl Verso enzyme mix, 1 μl (20 pmol) of forward primer IBV5 GU391 (5′-GCT TTT GAG CCT AGC GTT-3′) and 1 μl (20 pmol) of reverse primer IBV5 GL533 (5′-GCC ATG TTG TCA CTG TCT ATT G-3′), 0.25 μl of virus-specific probe (5′-FAM-CAC CAC CAG AAC CTG TCA CCT C-BHQ1-3′) (37) and 3.75 μl nuclease-free water. The thermal profile included a reverse transcription step at 50°C for 15 min followed by 15 min at 95°C. The PCR cycling was 40 cycles of denaturation at 95°C for 15 s, annealing at 60°C at 60 sec., and a final extension at 72°C for 10 min. To determine the IBV virus shedding titers, a standard curve was generated using titrated viruses in SPF eggs and the shedding titers were determined using interpolation (38).

### Histopathology

Tracheal and kidney tissues were collected at 7DPC and routinely processed, embedded in paraffin, cut into 5 μm sections, stained with hematoxylin and eosin, and examined under light microscopy. Lesion scoring was done according to Lee et al. (23). Briefly, tracheal epithelium deciliation and degeneration, glandular epithelium hyperplasia and hemorrhage were scored. Kidney lesions including tubular degeneration, renal nephrosis, necrosis and presence of renal casts, and hemorrhage were also scored as 0 for normal, 1 for focal lesions, 2 for multifocal lesions, and 3 for diffuse lesions.

### Statistical Analysis

The differences in ciliostasis scores, HI antibody titers, and virus shedding titers were estimated using One-way ANOVA with Tukey's *post-test* through GraphPad Prism version 5.00 for Windows (GraphPad Software, San Diego California USA).

## Results

### Genetic Analysis of the Vaccine Seed Viruses

**Figure 1:**
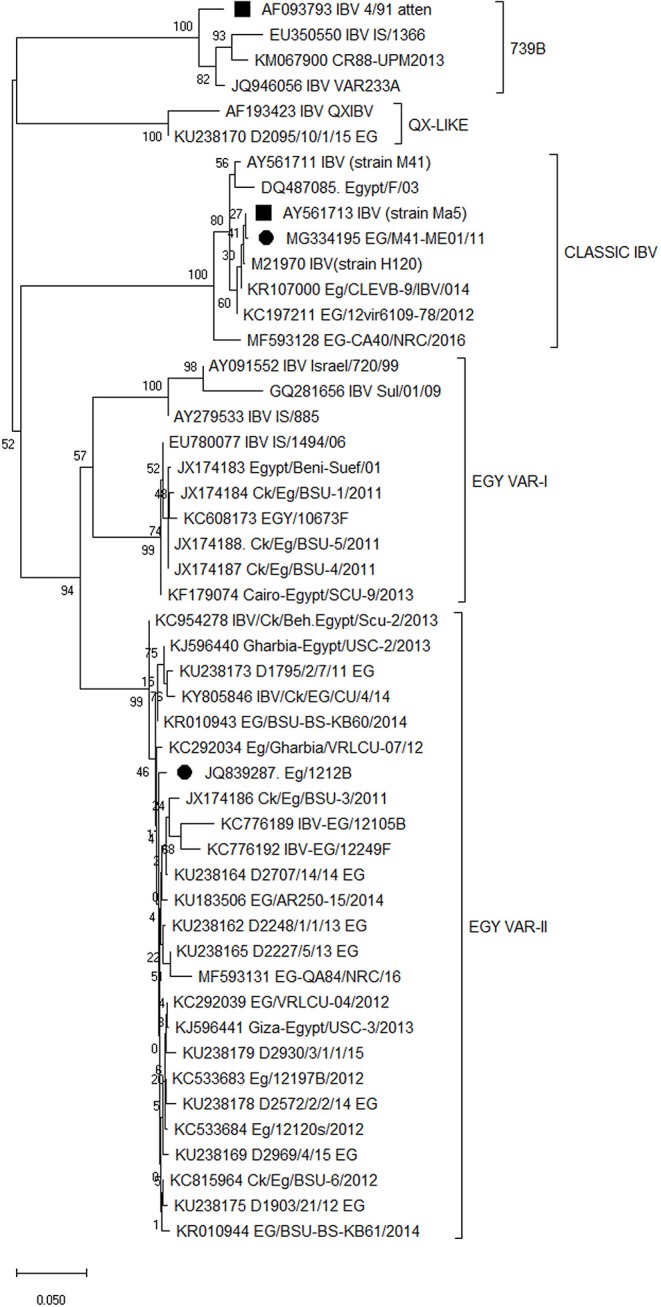
Full S1 gene sequence phylogeny of different vaccine seeds in relation to the available sequences on the GenBank. Vaccine seed strains are marked as 2 sets; middle eastern vaccine seeds (•) and imported vaccine seeds (■). Phylogenetic relationships through a bootstrap trial of 1,000 were determined with the MEGA version X using the Clustal W alignment algorithm and neighbor-joining method for tree construction.

The full S1 gene sequence alignment and phylogenetic analyses of different vaccine seeds showed that the predominant IBV strains circulating in Egypt are variant 2 strains belonging to the GI-23 lineage confined to and widely spread in the Middle East Region since its emergence in 2011. The variant 2 strains are distinct and the commercially available IBV vaccine protectotypes with high similarity to the Israeli variant strains IS/1494/06 and IS/885. The newly developed IB-VAR2 vaccine seed strain is genetically related to the currently circulating viruses in the region as compared to other vaccine seed strains (Figure 1).

### Safety of Different Live Attenuated IBV Vaccines in SPF Chickens

**Figure 2:**
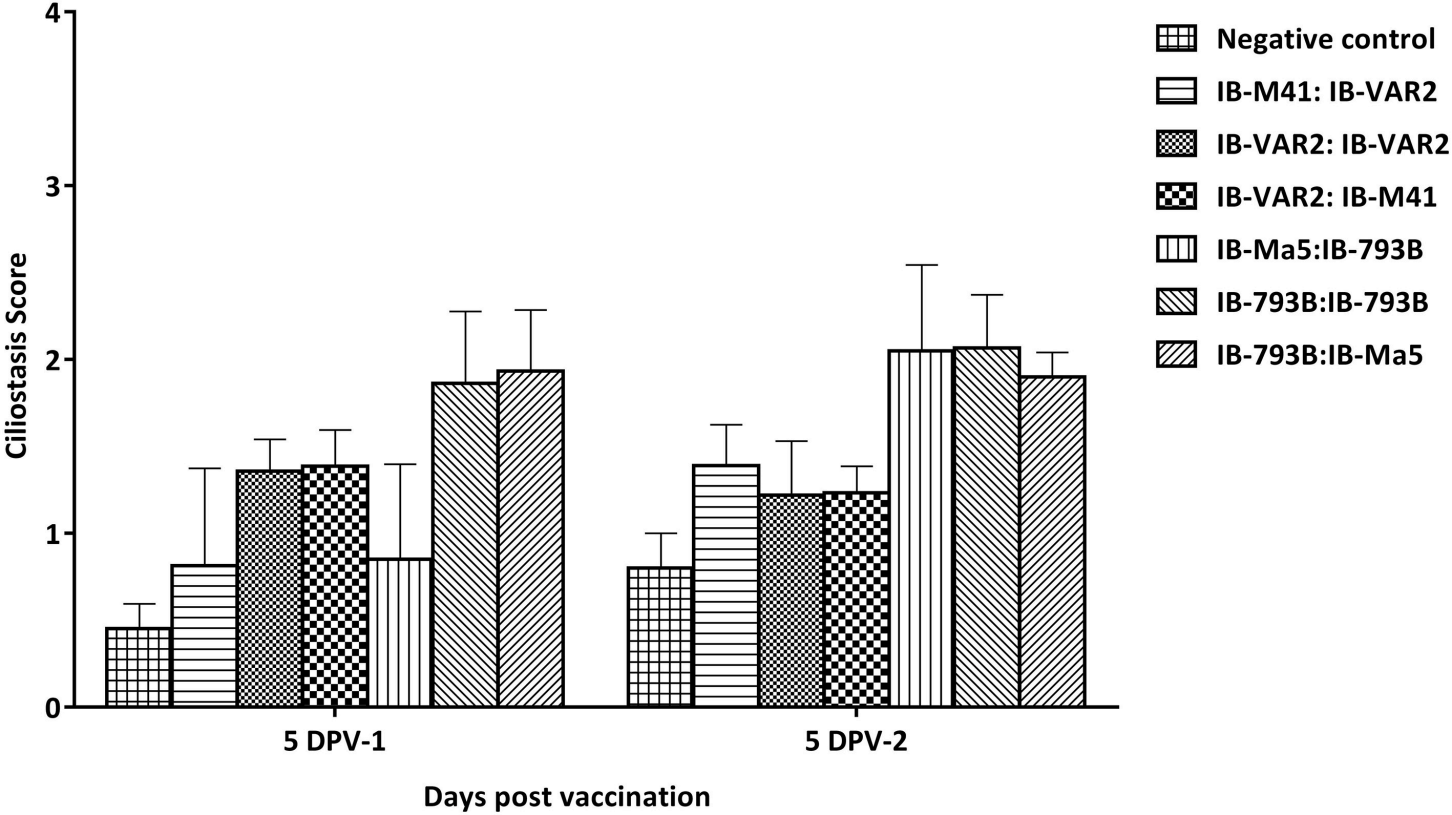
Ciliostasis scores in different vaccination regimes at 5 days post prime and booster vaccination.

All vaccines were well-tolerated in all vaccinated birds with all different vaccination programs. To confirm the safety of using different vaccination regimes, the ciliary activity in vaccinated chicks was assessed at 5 days post prime and booster vaccination. Results showed that vaccinated groups primed with IB-793B vaccine had slightly higher ciliostasis score (1.9 ± 0.3 compared to all other vaccinated groups. Similarly, the IB-793B boosted groups showed the highest ciliostasis scores among groups (2.1 ± 0.7) (Figure 2). The groups primed with IBV-VAR2 vaccine induced higher ciliostasis scores compared to those primed with classical IB-M41 vaccine (1.4 ± 0.14 and 0.9 ± 0.31, respectively) at 5 DPV.

### Protective Efficacy of Different Vaccination Regimes Against Wild-Type Virus Challenge

#### Clinical Observation, Seroconversion, and Gross Lesion Scores Vaccinated Birds

Birds in the non-vaccinated group which challenged with IBV-VAR2 (Eg/1212B/2012) showed typical clinical signs including huddling, ruffled feather, increased water intake, and slight watery diarrhea. No obvious clinical signs attributed to IBV infection were recorded in all vaccinated groups. On day 14 after prime vaccination, there were no significant differences in antibody titers between groups received different vaccines. However, at 28 days post vaccination, higher antibody titers were observed in groups with IB-VAR2 vaccine. At 14 DPC, a relatively increased seroconversion was noticed in groups received IB-Ma5 and IB-793B vaccines compared to those received IB-M41 and IB-VAR 2 vaccines (Supplementary Figure 1).

Tracheal and kidney lesions scores were higher in the non-vaccinated group challenged with IBV-VAR2 (Eg/1212B/2012) at 3, 5, and 7 DPC (2.3, 2.7, and 1.5 respectively). Lesions include punctuating hemorrhages and catarrhal exudates in throat and trachea. The kidneys were swollen with frequent urate deposits. No prominent gross lesions were observed in vaccinated and negative control groups (Data not shown).

#### Post-challenge Ciliary Activity

**Figure 3:**
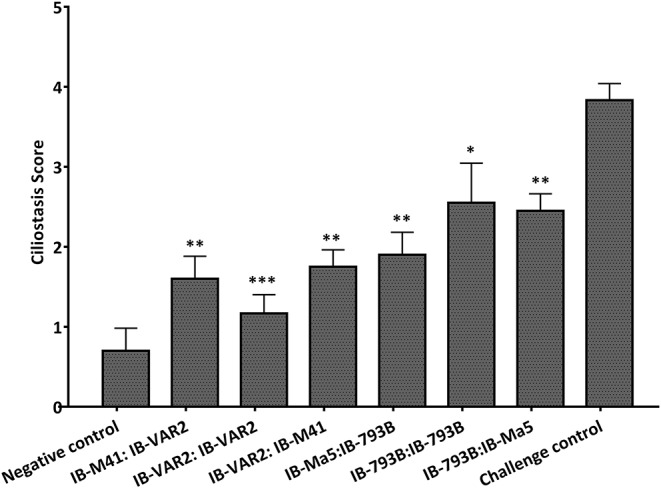
Ciliostasis scores in different vaccination regime groups at 7 days post challenge with Egy-Var 2 virus. DPV, days post vaccination; DPC, days post challenge. Asterisks indicate significant differences (***p ≤ 0.001, **p ≤ 0.01, *p ≤ 0.05).

The average ciliostasis score in vaccinated groups primed or boosted with IBV-VAR2 ranged between 1.2 ± 0.2 and 1.7 ± 0.2. Meanwhile, vaccinated groups primed or boosted with IBV-793B showed higher ciliostasis scores (Figure 3). The calculated protection percent in groups primed or boosted with IBV-VAR2 vaccine were 100, 100 and 80% in groups 1, 2, and 3, respectively. The groups 4, 5, and 6 primed or boosted with IBV-793B vaccine showed 60, 50, and 50% protection, respectively (Table 1).

#### Viral Shedding

The IBV shedding titers in vaccinated groups were monitored at 3, 5, and 7 DPC. There was a significant reduction in the IBV shedding titers at 3 and 5 DPC in all vaccinated groups. However, the significance was more in group 3 primed and boosted by IBV-VAR2 vaccine (*P*-value <0.001). In terms of the number of shedders, groups 1, 2, and 3 primed or boosted by IBV-VAR2 vaccine showed the lesser number of virus shedders compared to all groups at all-time points (Table 2).

**Table 2:** Virus shedding titers (EID50/ml) in different vaccination regime groups at 3, 5-, and 7-days post challenge using rRT-PCR.

**Vaccination regime (1:14 day old)**	**Virus shedding titers (EID50/ml)^a^**		
	**3DPC**	**5DPC**	**7DPC**
IB-M41: IB-Var2	2.05 ± 0.79^***^ (5/5)^b^	1.61 ± 0.39^**^ (4/5)	0.81 ± 0.31^*^ (2/5)
IB-Var2: IB-Var2	1.47 ± 0.20^***^ (4/5)	1.19 ± 0.26^***^ (4/5)	0.64 ± 0.54^*^ (2/5)
IB-Var2: IB-M41	1.95 ± 0.66^***^ (3/5)	1.60 ± 0.55^*^ (3/5)	1.16 ± 0.97 (3/5)
IB-Ma5: IB-793B	1.91 ± 0.68^***^ (5/5)	1.52 ± 0.67^**^ (5/5)	1.31 ± 0.64 (4/5)
IB-793B: IB-793B	3.12 ± 0.10^**^ (5/5)	1.73 ± 0.47^**^ (5/5)	1.25 ± 0.45 (3/5)
IB-793B: IB-Ma5	1.67 ± 0.19^***^ (5/5)	2.01 ± 0.48^*^ (5/5)	1.10 ± 0.77 (3/5)
Challenge control	4.07 ± 0.12 (5/5)	2.93 ± 0.61 (5/5)	1.90 ± 0.31 (5/5)
Negative control	0 (0/5)	0 (0/5)	0 (0/5)

a EID50, embryo infective dose fifty; DPC, days post challenge. Asterisks indicate significant differences to the challenge control group (^***^p ≤ 0.001, ^**^p ≤ 0.01, ^*^p ≤ 0.05).

b *Numbers in parenthesis indicate number of birds tested positive for virus shedding out of tested birds*.

#### Histopathology

Histopathologic lesion scores at 7DPC showed that the tracheas from birds primed or primed and boosted with IB-793B showed the highest lesion score among different vaccination regimes. Priming or boosting of birds with IB-Ma5 combined with IB-793B showed lower tracheal lesion scores. In kidneys, variable lesions scores were observed after challenge especially renal nephrosis. However, renal nephrosis was more severe in IB-Ma5 and IB-793B vaccines combination (Table 3).

**Table 3:** Tracheal and kidney histopathology lesion scores at 7 days post challenge.

**Vaccination regime (1:14 day old)**	**Trachea^a^**				**Kidney^a^**				
	**Deciliation**	**Epithelial degeneration**	**Glandular epithelium Hyperplasia**	**Hemorrhage**	**Degeneration**	**Necrosis**	**Renal nephrosis**	**Renal casts**	**Hemorrhage**
IB-M41: IB-Var2	0	0	1	0	1	0.5	0.3	0.5	0
IB-Var2: IB-Var2	0	0	1	0	1	0.5	0.5	1	1
IB-Var2: IB-M41	0	0	1	0	0	0.5	1	0	1
IB-Ma5: IB-793B	1	0.4	1	0	1	0.5	1.5	1	0
IB-793B: IB-793B	1.3	1.7	1	0	0	0.5	1.2	0	1
IB-793B: IB-Ma5	0.5	1	1	0	1	0.7	1.2	0	1
Challenge control	2.5	2.7	3	3	2.3	3	3	1	1
Negative control	0	0	0	0	0	0	0	0	0

a *Lesion scoring was done according to Lee et al. (23), kidney lesions were scored as 0 for normal, 1 for focal lesions, 2 for multifocal lesions, and 3 for diffuse lesions*.

## Discussion

Live attenuated vaccines are an important component of the vaccination program against IBV depending on their induction of mucosal immunity (19, 39). The continuous evolution of IBV in Egypt has reduced the efficacy of licensed vaccines considering that there is no protectotype identified for the IBV GI-23 lineage circulating in the Middle East (40). In this study, different combinations of live IBV vaccines with either homologous or heterologous variant IBV vaccines has been compared in SPF chickens.

Phylogenetic analysis reveals that the commercial vaccines IB-M41(Eg/11539F) and IB-VAR2 (Eg/1212B) vaccinal seeds are closely related to the classical Mass-like and variant 2 IBV (IS-1494/06 like) currently predominating in the Middle East region (Egypt, Israel, Iran, Saudi Arabia and UAE) (27, 29). These results further confirm the widespread of IBV strains belonging to the GI-23 lineage confined to the Middle East region (40).

All vaccinated birds with all different vaccination programs appeared healthy after both prime and boost vaccination. However, IBV-793B vaccine induced higher ciliostasis scores in primed or boosted groups, followed by groups vaccinated with IBV-VAR2 vaccine compared to those primed with classical IBV vaccines. Though, it has been widely accepted that attenuated IBV vaccine induce certain degree of ciliostasis depending on the degree of attenuation (19, 23, 41), the effect of IBV attenuated vaccines on tracheal ciliary activity has to be considered when measuring the vaccine protective efficacy (42, 43).

The HI test is a useful tool in monitoring the immune status of vaccinated birds, hence, the HI test was conducted using the IB-VAR2 antigen. Studies showed that sequential inoculation of different IBV may increase the broadness of the reacting antibody titers (44). This was true as indicated by the increase of the cross-reactivity of sera collected from groups received IB-Ma5 and IB-793B as prime or booster vaccination to IB-VAR2 antigen, however, it was significantly lower than homologous vaccination groups (i.e., IB-VAR2) at 28 days post vaccination. Additionally, the mean HI antibody titers in the heterologous variant IBV vaccine groups (i.e., IB-793B) showed a relatively higher increase at 14 DPC indicating the challenge virus replication in vaccinated birds. It is worthy to note that antisera of IBV H52, H120, and 793B failed to neutralize Iranian IBV genotypes including IS-1494, IS-720, 793/B, and IR-1 (45).

After the challenge of vaccinated birds, neither clinical signs nor obvious gross lesions were observed in all groups. However, variable ciliostasis scores were observed between vaccinated groups. The best protection in terms of ciliary activity was observed in groups primed or boosted with IBV-VAR2 (80–100% protection). Meanwhile, 50–60% protection was reported in groups primed or boosted with IBV-793B with significantly higher ciliary damage. This finding further emphasizes the importance of the homology of the IBV vaccine to the predominant field strains to provide the highest protective efficacy. Recent studies showed that vaccine candidates developed from Chinese QX-like IBV or Korean nephropathogenic IBV strains are better protective than heterologous vaccines (16, 21, 24).

A prime-boost vaccination program using IB-Ma5 and IB-793B provided satisfactory protection against IBV QX strain in SPF and commercial broiler chickens, however, about 50% of the commercial broilers were shedding the challenge QX strain with relatively high titers (22). Similarly, our results showed that the inclusion of IB-793B vaccine strain did not reduce the virus shedding and the homologous IBV-VAR2 vaccine primed and boosted groups have the most significant reduction in virus shedding titers (*P*-value <0.001) and the lower number of shedders. Though the IBV-793B vaccines have been advocated to induce heterologous protection, recent studies showed that the protection is mainly attributed to the relative virulence of the strain allowing it to displace other field strains after vaccine introduction (46). The inclusion of a vaccinated group primed and boosted with IB-793B which showed only 50% protection further support that relative protection afforded by IB-Ma5: IB-793B prime-boost programs is probably attributed to the effect of the Mass vaccine (47).

Though not studied, the cellular and local immune responses (i.e., CD4^+^, CD8^+^ IgA-bearing B cell expression) induced by IBV were suggested to be an explanation of heterologous protection of IB-Ma5 and 793B prime-boost vaccines. However, the cellular and local immune responses were found to positively correlate with the virulence of the vaccine strain (39). This correlation, despite enhancing protection, it greatly affects the suitability of such regimes in Egypt considering the exposure of vaccinated birds to multiple respiratory viral and/or bacterial pathogens interacting with IBV (2, 3, 48).

## Conclusions

To summarize, the current study confirmed that the efficacy of live attenuated IBV vaccines is more related to the genetic relatedness between the vaccine and field strains. However, prime-boost programs combining both classic and genetically related variant IBV vaccine strains are required to broaden the protection against different IBV strains field challenge. Cross-protection studies are essential besides determining the effect of vaccine strains on ciliary activity for both vaccine evaluation and proper design of vaccination program for IBV considering the field complication with other respiratory pathogens in Egypt.

## Data Availability Statement

The datasets generated for this study can be found in the Genbank.

## Ethics Statement

All experiments were conducted according to Animal Research Ethics Guidelines with approval from Faculty of Veterinary Medicine, Beni-Suef University, Egypt (17/03/2019).

## Author Contributions

HS, WE, and AA: conceptualization. AA, WE, AB, and WK: methodology. AA, WK, AB, and MZ: investigation. AA, WK, AB, and MZ: data analysis. WE and WK: resources. AA, WK, AB, and MZ: data curation. AA, WK, and AB: writing—original draft preparation. HS, AA, WE, AB, WK, and MZ: writing—review and editing.

### Conflict of Interest

The authors declare that the research was conducted in the absence of any commercial or financial relationships that could be construed as a potential conflict of interest.
