# Supplementary material for: Protective Efficacy of Different Live Attenuated Infectious Bronchitis Virus Vaccination Regimes Against Challenge With IBV Variant-2 Circulating in the Middle East
Source: Front Vet Sci. 2019 Oct 9;6:341. doi: 10.3389/fvets.2019.00341 (PMC6794438; doi:10.3389/fvets.2019.00341)
Supplement: Supplementary file 1 [file Presentation_1.PPTX]

## Slide 1
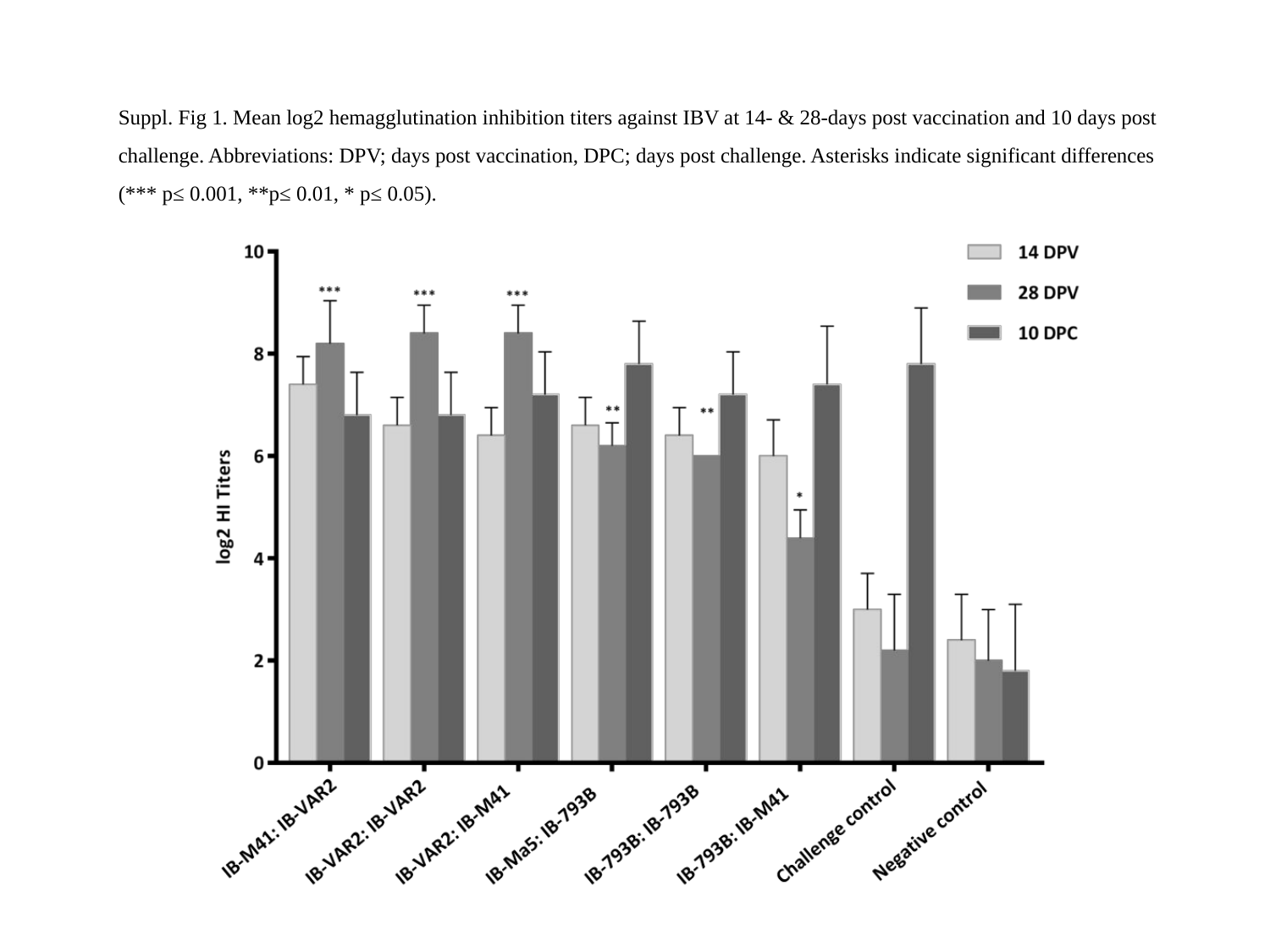

Suppl. Fig 1. Mean log2 hemagglutination inhibition titers against IBV at 14- & 28-days post vaccination and 10 days post challenge. Abbreviations: DPV; days post vaccination, DPC; days post challenge. Asterisks indicate significant differences (*** p≤ 0.001, **p≤ 0.01, * p≤ 0.05).
